# Environmentally Reformed Travel Habits During the 2006 Congestion Charge Trial in Stockholm—A Qualitative Study

**DOI:** 10.3390/ijerph8083202

**Published:** 2011-08-02

**Authors:** Greger Henriksson, Olle Hagman, Håkan Andréasson

**Affiliations:** 1 Department of Urban Planning and Environment, KTH Royal Institute of Technology, Drottning Kristinas väg 30, SE-100 44 Stockholm, Sweden; 2 Department of Sociology, University of Gothenburg, SE-405 30 Gothenburg, Sweden; E-Mail: olle.hagman@sts.gu.se; 3 Department of Cultural Sciences, University of Gothenburg, SE-405 30 Gothenburg, Sweden; E-Mail: hakan.andreasson@ethnology.gu.se

**Keywords:** everyday life, habits, sustainable travel, travel demand management, congestion charge, policy innovation, transportation

## Abstract

Policy measures that reduce or replace road traffic can improve environmental conditions in most large cities. In Stockholm a congestion charge was introduced during a test period in 2006. This was a full-scale trial that proved to meet its targets by reducing traffic crossing the inner city segment during rush hours by 20%. Emissions of carbon dioxide and particles were also substantially reduced. This study, based on in-depth interviews with 40 inhabitants, analyses how and why new travel habits emerged. The results show that particular, sometimes unexpected, features of everyday life (habits, resources, opportunities, values, *etc.*) were crucial for adjustment of travel behaviour in relation to the policy instrument. One example was that those accustomed to mixing different modes of transport on a daily basis more easily adapted their travel in the targeted way. On a more general level, the results revealed that the policy measure could actually tip the scales for the individual towards trying out a new behaviour.

## 1. Introduction

Traffic in cities has negative effects on health and the environment. The more congested the traffic and the larger the proportion of car traffic, the worse these effects seem to be. The effects can be reduced by traffic reduction, or by shifting from cars to other modes of transport with less negative impacts, such as public transport. Measures taken in order to reduce car traffic are often unpopular and their implementation met with resistance. However, in some cases they can be successful both in terms of public opinion and environmental effects. Today a number of cities are considering the implementation of radical policy measures and the congestion charging in London has become a “global prototype”. Though not very popular among motorists, congestion charging has the potential to appeal to such diverse interests as classical economists that favour a user pay model, businesses that suffer from reduced accessibility, environmental groups that want reduced air pollution, and social justice activists that want improvements in public transport. Besides, since London is recognised as a world city and a forerunner in urban modernisation, any policy successfully implemented there has great symbolic value [1,2].

This paper is based on two in-depth ethnological studies of choices and reactions in relation to the congestion charge that was implemented in Stockholm in 2006 [3,4]. The background to the introduction of congestion charging in Stockholm was that after the Swedish general election in 2002, the Social Democrat, Green and Left political parties promised to make a “test” introduction in Stockholm before the next election in 2006. After a series of problems, concerning the procurement of technology, the political process in itself *etc.*, the test finally took place. Congestion charging, in the form of a tax administered by the state, was introduced from January to July 2006, but during the whole trial period from September 2005 to December 2006 public transportation was enhanced through the purchase of nearly 200 new buses, used to intensify traffic on existing bus lines and to open 16 new lines. Most of these were non-stop lines between the suburbs and the city centre during peak hours, running parallel to the underground or commuter trains. Together with a few other minor measures, e.g., some new park-and-ride locations, this was officially known as the Stockholm Trial (below also referred to as “the trial”), with a total budget of about 400 million Euros. The congestion charge varied during week-days from 10 SEK (≈1 Euro) to 20 SEK for each passage in or out of the zone. The maximum fee was 60 SEK per day and vehicle. There was no charging at nights, or during weekends or holidays [5].

The congestion charge was aimed at reducing the negative impacts of car traffic, in order to “reduce congestion, increase accessibility and improve the environment” [5]. The evaluations included measurements of traffic flows, congestion, and also levels of carbon dioxide, particles, nitrogen dioxide and noise [6]. In terms of documented effects, in 2006 the congestion charge in Stockholm decreased car traffic to and from the inner city by 20% during rush hours; increased the proportion of “green cars” exempt from congestion charging (hybrids, electric, ethanol, *etc.*); reduced health related emission (particles, *etc*.) in and around the inner city; and gave a substantial decrease in greenhouse gas emissions, to have been achieved through one single measure, all according to the overall official evaluation of the trial [6].

The congestion charge was evaluated during the trial period and two months after it, at which point there was a referendum to decide whether it should become permanent or not. In this way the trial provided social scientists with the opportunity to study how a temporary change in conditions might influence travel. It turned out that the seven-month period was long enough to observe substantial effects, and these effects were documented by quite a few researchers, studying, e.g., travel patterns and attitudes. One important observation was that more people became positive to congestion charging during the test period and that this was due to their personal experience of effects of the measures introduced [7]. It was also concluded that the increase in support was related to there being a high proportion of public transport users in Stockholm [8]. This increased support was somewhat related to the expansion of public transport services during the trial [8]. Quantitative methodologies and large samples were mostly used to reach conclusions of this kind, which makes them reliable on the aggregated level. However, to give a socio-cultural understanding of the mechanisms behind the responses, *i.e*., how and why travel habits were adapted, qualitative methodologies were also needed. Such methodologies were applied to a much lesser extent and to our knowledge only the ethnographic studies by the authors were performed during the Stockholm trial [3,9]. In our view the novel contribution of this study to the overall body of social science studies on the Stockholm trial is the use of ethnographic methodology. This methodological approach has a clear focus on socio-cultural and situational aspects.

The general aim of this study was to analyse how the Stockholm trial influenced travel habits and opinions, with the focus on environmentally significant choices. Our investigations were carried out using qualitative methodology at the individual and household level in order to supplement the more abundant quantitative surveys reporting on effects at the aggregated level. The specific aim of this study was to characterise social factors that possibly tipped the balance for the studied individuals concerning (i) their choices of whether and how to adapt their travel in the targeted way; and (ii) changes in their opinion of the policy instrument.

### 1.1. Methodology

The methodological approach used in this study was built on the well-established “activity approach” [10]. This approach viewed travel as a complex phenomenon, depending on the various needs and demands of individual users that justify their activities, and on the potential and opportunities they have to fulfil them. Both objective and subjective factors affecting travel behaviour were taken into account. The method also took a “situational approach” to transportation patterns, which means that individual trips were seen as parts of a “highly complex series of interrelationships of various trips, in-home, out-of-home and household activities” [10,11]. A qualitative method is necessary to understand how changing conditions, such as the introduction of a congestion charge, can lead to changes in such complex interrelationships.

A basic assumption in the study is that transport is a constituent of everyday life. It is through transportation, physical or virtual, that humans connect to other people, and get access to different resources and activities. Transportation is interwoven with everyday life and commitments [12–14].

Today’s travellers are members of social communities that stretch beyond their local neighbourhoods, and they are dependent on technological artefacts and infrastructures to maintain their activities [15]. This means that the everyday life is a socio-technical project. In order to gain access to resources, activities and other people situated outside their area of residence, people use technology and financial resources in relation to their social relations and cultural values [16].

Depending on the role of cars in their everyday travel, through choices of lifestyle and habitation, and depending on economic resources, three different types of everyday travellers in the city can be defined:

- “Habitual car-users” seldom use any other means of transport than the car and they are dependent on it for most of their everyday trips.
- “Mixed mode users” have organised their everyday travel to include regular use of more than one means of transport.
- “Habitual public transport users” use public transport for most of their everyday trips.

Mixed mode users and even habitual public transport users may be more or less dependent on cars for a few but essential parts of their everyday travel, such as weekly shopping for non-durables. Only very few people are “car-free”, which means that they have in some way or other, by their own will, managed to become independent of the private car. They should not be confused with the “car-less”, who have to manage without cars against their own will [17]. It has also been shown that mixed mode users and habitual public transport users often deliberately chose to live close to public transport services and have incorporated these among their resources [17].

In order to be able to connect the choices made by the individuals to their motives and commitments, the present analysis was based on in-depth, semi-structured interviews with 40 persons in and around Stockholm. The first 20 of them were interviewed twice, during and after the trial [3]. The second 20 of them were asked to fill in a travel diary before and during the trial [9]. In the 12 cases where this request was fulfilled the first diary was used in the interview to ask questions about motives and commitments behind particular journeys. In addition to this, a number of shorter interviews were held on-the-spot in traffic environments such as bus stops, petrol stations and park and ride lots.

In the following is briefly presented who were recruited for the interviews and how this was done. It should be noted that the selection of interviewees was entirely aimed at providing a satisfactorily material for a qualitative method and analysis. The selection should not be regarded as representative in a statistical sense. However, to provide the reader with some opportunity to relate our study to the quantitative ones it is outlined below features of our sample such as age, place of residence and most commonly used means of transport.

The first 20 interviewees were recruited by the authors who did a random sample of people with residence in three recently built neighborhoods. Subjects were given a letter describing the survey’s purpose and structure and were then called to make an appointment for an interview. The selection from three newly built such areas was made on the presumption that they as newcomers would be more inclined to reflect on their travel habits since these would be relatively recently reestablished. Choosing the three neighbourhoods also provided the possibility to assess the importance of local contextual factors. The first neighbourhood was Hammarby Sjöstad (Hammarby Waterfront), a suburban area with multi-family houses, located just south of the charging zone and the proper inner city. The connections by public transport to the city centre are very good, and the area was designed for “eco-friendly” living. The second area was a suburban area of terraced houses located in Hallunda south of the centre of Stockholm. It has motorway and underground train connections to the city centre, and during the trial period these were complemented with new non-stop bus lines at peak hours. The third area was Bällstaberg in Vallentuna, a peripheral suburb north of the city, with a mix of terraced houses and multi-family houses. Bällstaberg has commuter train connections to the city centre, and these were also complemented during the trial period with non-stop bus lines.

The second 20 interviewees were recruited by the staff of the Stockholm City Museum. (The Museum also archived the interviews in the form of audio recordings and transcripts.) In recruiting the interviewees the museum staff used their contacts and a set of criteria. This meant that there should be interviews of people who met each of the following characteristics:

- Commuters by car who intended to continue with this during the trial, respectively who intended to switch to public transport.
- A few professional drivers (taxis, goods).
- People who disposed of own respectively of company cars.
- Commuters by public transport respectively users of mixed modes.
- Persons who stated a positive, negative respectively undecided attitude towards the trial and congestion charging.
- Members of different household/family types (singles, cohabitees, with/without children).
- Residents of inner city, suburbs respectively peripheral suburbs.

Females and males became about equally represented and the age distribution ranged from about 20 to 70 years. The distribution along this range was even except for an overrepresentation of (15% *extra*) persons in their 50s. They were selected because they volunteered at a shorter notice than other requested respondents and were thus able to fill in a travel diary *before* the trial. Among the interviewees roughly 50% were habitual drivers, 35% were mixed mode users, and 15% were habitual public transport users. This mirrors the conditions in greater Stockholm quite well. Three interviewees lived inside the charging zone and commuted to workplaces (or equivalent) outside of it. The remaining 37 interviewees lived outside the charging zone. Twelve of them commuted to workplaces outside the zone (in six cases, *through* the zone*)*. The majority, 25 interviewees, commuted from outside the zone to workplaces on the inside. In terms of distance from the centre of Stockholm we have defined three types of areas, inner city (3 interviewees lived there), suburbs within 20 km (27 interviewees) and peripheral suburbs (10 interviewees).

## 2. Results from Interviews

Results from the interviews in accordance with the aim and methodological framework are presented below. This includes individual examples of ways in which travel was influenced by the congestion charge trial. (In the presentation below all persons are given false names.) Significant examples of how travel choices were influenced by the trial included individuals that did not change their travel at all, nor their acceptance of the charge. At one end of the spectrum were inveterate motorists that opposed the charge, and at the other end public transport users that were in favour of it.

### 2.1. Unchanged Motorists

Sten who lives in Hammarby Sjöstad, was very much against the trial, although he did not pay any charges himself since he commuted by car on the Essingeleden bypass road, which was exempt from the charge. In his view the traffic that had *moved* there had made it “totally congested”. He also said that his wife, who commuted by public transport suffered from increased crowding on trains/buses. In his opinion congestion charging was “just a stupid idea from the politicians”, “a waste of money” and he couldn’t in his “wildest fantasy imagine that we will vote for this”. Sten said that he had chosen to live quite far, “at driving distance” from his job, which meant he spent more than one hour a day commuting in his car. To him public transport was not an alternative. He said he would never use the underground in rush hour because there were so many people on it, “with their smells and all that”.

In our analysis we relate Sten’s negative view on the congestion charge to his experience of the car as a prime means of getting access to resources. In terms of cultural values, he adhered to a view of the car as an indispensable good. The congestion charge, aimed at reducing the use and utility of cars, challenged his view of the car, even if it did not affect his own driving, at least not for the time being.

In contrast, another unchanged motorist was Benny, who also lived in Bällstaberg. He regularly drove cross the charging cordon and chose to pay. Benny found it worth the money because the traffic situation became so much better. He said that when he drove his daughter to school one day “it was just like a Sunday morning”.

About half of the interviewees were habitual car users like Sten and Benny, and most of them expressed views like Sten’s on the congestion charge and the role of the car in their everyday lives. The interviewees also included habitual users of public transport. Their habits and opinions were of course different from the start.

### 2.2. Unaffected Commuters on Public Transport

One example was Rita, who lives in a rural area some 30 km north-east of Stockholm with her husband and teenage son. She commuted to and from work in Stockholm by bus. The bus stop was in the village of Brottby, a few kilometres from their home, and the family went there by car.

“In the morning we time our journeys to fit in with [our son] Rikard, who has to go to school, and in the evenings everybody fits in with me, because I have to travel a bit further than anybody else. So we all meet in Brottby at half past five. Sometimes Rikard gets there about four and then he might catch the last school bus all the way home. [...] And besides, it’s so fantastic that in Brottby there’s a library next to the grocery store that’s open for as long as the store is. [...] I usually sit there reading newspapers. It’s a nice place to be, very convenient for commuters. It doesn’t matter if anybody’s late. We have the library as our meeting place. [And then] we generally do a bit of shopping at the supermarket – some milk, that kind of things.”

The above demonstrates how the activities of the family members are synchronised, and their journeys routinized in relation to this. Factors such as working hours, travel times and school timetables are managed. If Rita had used a car, she could have fitted in more journeys per day or per week. However Rita was accustomed to using public transport; and that combined with her rural location imposed restrictions on the extent to which she wanted to travel. An interesting point was how these circumstances were used positively, and how even waiting times can be given a positive spin. This also points to how established routines are of importance when it comes to how people react and adapt if conditions change.

Like several other interviewees who did not have access to a car, Rita said she had not changed her travelling habits during the trial, and her travel diary confirmed this. She had not changed her general opinion of the congestion charge either, but said she had been in favour all along, since she was simply “one of those who ride a bicycle and go by public transport”. Her attitude had become more positive during the trial because of the great impact she had observed on traffic. “I’m actually pleasantly surprised, I must say”. She also mentioned the advantages of better environment and air in Stockholm and fewer delays on buses.

Another who did not change, because the new conditions suited her well, was Sylvia, a woman in her fifties living in Hammarby Sjöstad together with a partner about the same age and his teenage daughter. They had no car. Sylvia walked to the shopping mall, which was about 500 m away, to buy food and the like. She said she needed to go for a walk anyway, just to get out, because she worked a lot from home. The shopping was an almost daily routine, so her bags never got too heavy. Thus, these walks were not only for shopping, but also included getting exercise as well as social motives. Sylvia said she loved public transport, especially the ferry boat going across the cove to the inner city, which was a “delight to travel on”. She was pleased that the congestion charge trial had turned out so well, and that traffic decreased without any chaos. Before the charge was introduced she said it would “make people start to think”, indicating that many people use the car habitually and need help to break this habit. However, she did not think people would welcome this help. “One must expect an outcry in the beginning, but then it turns out that it was not a bad thing”. She also said “it is nice that Stockholm is in the front-line of development”.

We observed that in general the users of public transport kept a lower profile in their views in favour of the charges, compared to how the motorists in most cases emphasized their strongly negative attitudes and the reasons behind these. The reason for this observed difference might be that those in favour had no real need to demonstrate their support. They had, at least temporarily, the powers that be on their side. Many of those who were critical, however, felt compelled from the very start to demonstrate what they thought.

Concerning travel habits it is revealing how daily activities, culturally grounded values and other elements in their daily lives held the travel of the unchanged in place, so to speak. In the case of the motorist Sten, his commuting route as well as his car-related values could be seen as elements of his travel network that stabilised his habits in relation to the charges. Correspondingly, in Rita’s case, values related to family, use of leisure time, rural place of residence and a firmly established public transport routine seem significant. These elements made her favour the charge.

### 2.3. Intentions to Resist and to Change

All interviewees were asked if they would do, or had already done, anything about their travel in relation to the introduction of the charge. Most motorists said that their intention was to resist in one way or another. A few persons that were largely unaffected, like Sten above, claimed they wouldn’t change, but carry on driving as before. A taxi driver in his 60s remarked, when interviewed on the spot on the first day of the trial, that his taxi was exempt from the congestion charge and continued: “Our private car belongs to my wife, and that car isn’t going to adapt at all. Wherever she needed to drive before, she will continue to drive now”.

However, most motorists were affected for at least some of their journeys. The most common response among affected motorists before, or during the first months of the charge was the intention to try to stay out of the whole business because of a deeply felt dislike of it. This in turn led to the intention not to pay. Quite a few said this would be achieved by not driving wherever and whenever charges applied. One example was Tina, a middle-aged civil servant who lived in Täby and worked in the city centre: “I can tell you what I thought. [...] I’d more or less made up my mind to leave the car at home and use public transport”. This specific kind of attitude and intention is also illustrated by a quote from a woman called Cecilia: “I more or less refuse to even try to pay this congestion charge. And so I haven’t used my car. [...] I suppose that’s the result they [politicians] want to see, of course. But even so, I don’t think it’s the right way to go”. And also by Hanna: “this is the very first time I’ve been forced to pay the congestion charge. [...] I’d made up my mind that I wasn’t going to do it. I’d intended to avoid paying”.

If the travelers were put on an imaginary seesaw (or balance) those who stopped driving would make the seesaw tip in the direction of reduced traffic and congestion. This represented a paradox or a dilemma for the motorists who intended to avoid driving whenever charges applied, as they would still be counted as a positive outcome in terms of reduced traffic.

However, it turned out that some of these motorists paid up anyway, and continued to drive. Tina said: “Before it even occurred to me that I ought to continue driving and pay the charges [...] I remember saying to my husband ‘I’m a bit worried about how long it’s going to take and what effect that will have, especially as I often work late. It’s just not possible to get home […] in the late evening’. ‘Yes, I suppose you’d better pay up, then’, he said. ‘If you think it’s important to get to work and back home again quickly, the best thing for you to do is to pay the congestion charges’. And he was quite right, of course. But that wasn’t the way I was thinking to start off with”.

The adaptability shown in this case is that Tina was able to change her mind, pay the charge and carry on driving to work under the new conditions she felt were imposed on her. To adapt her thinking she relied, among other things, on her husband’s opinion. This illustrates how social resources could be used to develop a strategy for how to manage one’s travel under changed conditions, and also how culturally grounded standpoints were negotiated.

Tina changed her first decision. Some others, however, stuck to their decision not to drive. This applies for Cecilia and Hanna, quoted above. They mainly managed to stay out of the system and not have their number plates registered by it, but in the traffic counts they were of course counted as cancelled trips or reduced traffic. In relation to the objectives of this study, drivers like Hanna and Cecilia changed their travel. They did not necessarily feel that this was the ultimate choice in terms of demonstrating their standpoint, but it was the one choice they felt they were left with. Taking the seesaw metaphor further, it can be said that they tried to jump off, but that this actually made it tip in favour of the charges. On the other hand, those who chose to carry on driving exactly as before and pay the charges might well also have felt that they had surrendered and obeyed the instructions issued by the powers that be.

This shows that one factor that might be decisive was a personal urge to stay out of the whole business. But who was able to stick to this tactic? Interviews and travel diaries showed that one characteristic was using mixed modes of transport, at least for trips to the city centre. Hanna and Cecilia, for instance, drove into the zone a few times per month before the trial. That might have helped feeling they could *afford* to change a few trips. Anyway, for the analytical purposes of this paper it meant that mixed users turned out to be an interesting category.

A lot of smaller changes were made by mixed users, including those who were ambivalent or in favour of the charges. They changed a few of their car trips, e.g., for shopping or leisure and sport activities, to biking or public transport, or they changed destinations so that they did not have to pass through the charging cordon.

### 2.4. Those Who Adjusted Their Travel Routines

Karin in Hallunda changed from the underground to the new bus. During the trial she found that the new bus line often got stuck in traffic on Fridays. Although it was sometimes delayed, she still regarded it a substantial improvement. She could sit on the bus without having to crowd with a lot of people, as she had to do on the underground. It was not faster, but less crowded. It was her dislike of crowding that triggered her change and she hoped that not too many would discover the new bus line, because then the advantage would disappear. Karin adjusted her travel mode due to the trial, although it was not the charge but the new bus line that made her change. It solved a problem she had experienced for a long time, crowding on the underground.

Kalle was a young mixed mode transport user in Hallunda who changed his driving routines slightly during the congestion charge trial. Even before the trial his opinion was that one should use public transport instead of the car when going into the city, but that did not stop him from taking the car now and then to his work in the city “for convenience”. During the whole charging period he never used the car on these trips, “as a matter of principle”. The charge took away some of the convenience of the car that had previously seduced him into using it, and thus it helped him to act more in line with his own convictions.

Among our interviewees were several who adjusted their travel times or routes, avoided occasional trips, *etc*. Such adjustments included leaving home or work earlier or later to pay lower or no congestion charge. They also included occasional car trips being either cancelled or replaced, trip sharing with a spouse, working from home, using public transport instead of the car for occasional leisure time errands in the city, *etc*. The kind of motives given for such adjustments were (of course) to pay less congestion charge, but also to meet demands from employers, avoid particular traffic situations, save time, *etc*.

One person who changed his route instead of his travel mode was Bertil in Bällstaberg. He was a habitual car user who went by car every day to his work in Hammarby, on the opposite side of the congestion charge area. During the trial period he made a detour around the charge zone every day to avoid the charges, although this cost him time.

### 2.5. Major Changes

One person who more drastically changed his travel mode for commuting was Dennis. He was in his 50s, worked as an accountant and lived in Djursholm, some ten kilometres north-east of the inner city. Before the trial he nearly always drove his car to work in the city centre. During the trial, however, he decided to use public transport about four days a week. He explained that his going over to public transport had to do with his working hours:

“I often used to stay on working very late and thought it was OK working as long as that, 70 h a week sometimes. It was often gone 10 when I got home. [...] But a few years ago I decided to start reducing my working hours. Then I really brought matters to a head last year [2005]. One of the main factors was trying to use public transport a bit more often. That makes you more disciplined. [...] It becomes easier to observe specific times, and maybe only work overtime two days a week. [...] The extra cost was one of the reasons why I changed my habits. I used to think in terms of 60 SEK a day, even if it might only have been 40, or even 20 come to that. [...] And then it wasn’t all that much more comfortable to use the car. So I thought I might just as well use public transport, and when you do, you find it has quite a few advantages.”

Dennis’s way of thinking illustrates that for an individual, very specific and unexpected elements related to travel in his/her everyday life can matter in a specific situation. In this case a more than year-long personal effort to reduce working hours was made to interact with responding to the introduction of the charge. Dennis’s explanation of his motives behind the change also illustrates particular motivating needs and demands, and how the potential and opportunities to fulfil them can arise with changing conditions. Interestingly it was the flexibility of the car, so praised by most habitual car users, that he wanted to get away from, as he hoped that the less flexible commuter train would help him become more disciplined.

The variety of motives behind what are here referred to as marginal adjustments or major changes show that certain kinds of discrete tipping points in the individual travel networks were activated by the introduction of the congestion charge, and they could sometimes be activated in quite unexpected ways.

## 3. Discussion and Conclusions

An important environmental aspect of the trial was that a sufficient amount of people adapted their travel in the targeted way. This gave effects such as significantly less rush hour congestion, which was noticed by many citizens and affected their opinions [18]. Official polls and the referendum in 2006 showed that enough people changed their opinion from negative to positive during the trial. This implies that the adaptability among citizens, both in terms of travel behaviour and readiness to change their view, was crucial for the outcome.

What was it that made Stockholmers adjust their behaviour and attitude in relation to the congestion charge? This study showed that factors in everyday life, such as resources, opportunities and values, seem important. Examples of factors related to opportunities and values were “getting exercise” and “managing stress”. If e.g., switching from car to public transport for commuting is seen as a means to reduce stress, the policy measure could, in some cases, tip the balance for individuals and make them actually try out the new behaviour. This implies that methodological tools for understanding how different factors interact in everyday lives are needed. In the introduction section, concepts related to using and getting access to different social and technical resources were mentioned briefly. In the light of our investigation, this kind of framework seems useful for further studies and analyses.

It is obvious that the travellers in a large city like Stockholm are “networked travellers” in the dual meaning of the word [15]. Firstly their social networks are dispersed and complex in terms of coordinating activities and contacts with other people in space and time, and secondly they are dependent on technological networks and infrastructures to maintain their social networks. Thus, the everyday networks that they build are heterogeneous and include both humans and non-humans [19,20]. Everyday life is a socio-technical project and, when seen from the viewpoint of the home as a base for travel, it is also a “residing project”. People manage their residing projects through assembling networks that include everything from technology and financial resources to social relations and cultural values [16]. How successful people are depends on how they manage to create complete and smooth everyday networks, and on how they manage to stabilise and maintain these. The elements of these networks may be more or less co-operative. As an example, congestion charging was experienced as inconvenient in the same way as parking restrictions, and in many cases called for re-negotiation of the role of the car in the network, so that it was, e.g., replaced by public transport on trips to the city centre.

We also found that travel habits were mostly only slightly adjusted. Individuals replaced, re-scheduled or cancelled occasional trips. Official statistics from the trial show that such marginal adjustments still contributed significantly to the positive environmental effects on the aggregated level. This means that since adjustments on the individual level of travel, as well as in attitude, daily schedules, activities, *etc*. were relatively small, quite a low degree of flexibility in the individual networks seems to have been sufficient for the relative success of the reform.

Successful implementation of congestion charges can improve environmental, health and traffic conditions in a large city. Our results indicate that the acceptance and effectiveness of the congestion charge in Stockholm were related to certain forms of flexibility and robustness on the part of the citizens affected, viewed as networked travellers. Changes in public opinion over the course of the trial seemed to be related to the character of their residing projects and travel habits. In short, this means that an analysis based on the activity approach, applying the concepts of networked travellers and residing projects can contribute to the understanding of the prerequisites for a successful reform.

We identified a number of circumstances and conditions in the interviewees’ everyday routines (*i.e*., in their established travel networks and residing projects) which influenced (how they reacted and adapted) whether they were likely to change their travel habits in response to the charge. An important condition was that mixed mode users more easily changed (parts of) their travel habits. One reason for this was that many of them were already accustomed to making a conscious choice of travel mode before an individual trip. Another aspect was that if they had a habit and/or a conscious will to use a particular travel mode on a regular basis, this could influence choices of where to live, *i.e.*, close to public transport connections. Consequently, it could be easier to introduce a measure of this kind in a city where there is a sufficiently large proportion of travellers that use public transport and mixed modes. In the county of Stockholm, mixed mode users constituted 35–40% [21], and among those travelling across the charging cordon, surrounding central Stockholm, the proportion was even higher. For the outcome of the trial in terms of changed travel behaviour, they probably represented an important group. While it is easier to win over mixed mode users, it can be more difficult with habitual motorists. They are often ideologically in favour of the car and opposed to public transport. Many of them have never integrated public transport into their everyday lives, and therefore accessibility to public transport is often a non-issue in their choice of habitation.

The variety of causes and motives behind the environmentally important travel choices show that certain kinds of discrete tipping points in the individual travel networks were activated by the introduction of the congestion charge, and that the scales could sometimes be tipped for quite diverse and unexpected reasons.

However, it should be noted that the importance of elements and flexibility in everyday networks in general, and discrete tipping points in particular, is not something we had anticipated before performing our study. It was only afterwards, when trying to explain the (unexpected) success of the reform, that we developed this perspective. This, in turn, means that we would like to see new qualitative studies of adaptation to congestion charging and other environmental policy instruments, so that the perspective can be further tested.

If we were to make any recommendation concerning the introduction of congestion charges, it would be of a general kind: If, in the city in question, there is: (i) a politically decided environmental objective to reduce car traffic, emissions and/or congestion; (ii) a political window of opportunity for this; and (iii) public transport/mixed mode users and public transport capacity to roughly the same extent as was the case in Stockholm, congestion charging should be tried, continuously evaluated (especially during the first year) and perhaps voted upon. A slightly negative public opinion should not be too great an initial deterrent, since substantial positive effects on congestion and the environment could alter the opinion. Under such conditions, with some flexibility on the everyday life level, admitting tipping points, the chances are good that congestion charges, if introduced, would become accepted and efficient. It is also possible that similar measures, e.g., environmental zones, could be implemented successfully in relation to a similar flexibility and adaptability in inhabitants’ everyday life and travel networks.

Finally, the story of the trial ended with a yes vote. A slight majority of those entitled to vote (inhabitants in the City of Stockholm, but not in the rest of the county) said yes to the congestion charge in the referendum of 2006. This led to the charge being reintroduced on a permanent basis from 2007. The support for the congestion charge continued to increase after its reintroduction in 2007 [22]. Conditions regarding congestion and emissions are still better than before the trial, but slightly worse than during it. An adjustment of the congestion charge level, especially during rush hours, might reduce congestion and emissions once again. Such a cost increase may be received with greater acceptance if it is once again accompanied by other measures such as improvements to public transport, cycling conditions and/or tax reductions in other policy areas.
